# HER2 heterogeneity and resistance to anti-HER2 antibody-drug conjugates

**DOI:** 10.1186/s13058-020-1252-7

**Published:** 2020-01-31

**Authors:** Alberto Ocaña, Eitan Amir, Atanasio Pandiella

**Affiliations:** 1 Experimental Therapeutics Unit, Medical Oncology Department, Hospital Clínico San Carlos and IdISSC, Madrid, Spain; 2 Centro de Investigación Biomédica en Red Cáncer (CIBERONC), Madrid, Spain; 3 Centro Regional de Investigaciones Biomédicas, Castilla-La Mancha University (UCLM), Albacete, Spain; 4 Division of Medical Oncology & Hematology, Department of Medicine, Princess Margaret Cancer Centre and the University of Toronto, Toronto, ON Canada; 5 IBMCC and IBSAL, Salamanca, Spain

**Keywords:** HER2 heterogeneity, HER2 resistance, ADCs, T-DM1, DS-8201

## Abstract

**Background:**

There has been substantial interest in HER2 intratumoral heterogeneity as an explanation for the development of resistance to anti-HER2 therapies in breast cancer, particularly to trastuzumab emtansine (T-DM1).

**Methods:**

Through a literature-based approach, we discuss mechanisms of resistance to HER2-targeting antibody-drug conjugates (ADCs) in breast cancer.

**Results:**

We describe results from clinical studies reporting the effect of anti-HER2 strategies particularly ADCs and their mechanistic effect. We review biological findings underlying HER2 heterogeneity and its implication in the development of novel anti-HER2 drugs including new ADCs in clinical development like trastuzumab deruxtecan (DS-8201).

**Conclusions:**

We suggest potential mechanisms to optimize these compounds and their future clinical implementation.

## Tumor heterogeneity and HER2 expression

Oncogenic transformation is mediated typically by the sequential accumulation of genomic alterations in non-transformed cells [1]. These changes occur over time and modify biological functions including survival, proliferation, migration, or invasion, among others [1]. Genomic analyses have demonstrated that tumor heterogeneity can occur due to the presence of clones with different characteristics, and such molecular heterogeneity may explain adaptation in response to treatment exposure, leading to drug resistance [1].

HER2 is a transmembrane tyrosine kinase protein that belongs to the ErbB family of receptor tyrosine kinases [2]. In some solid tumors, the *HER2/neu* gene is amplified leading to an increased expression of the HER2 protein [2]. Tumors with such amplification include, among others, breast, gastric, bladder, and non-small cell lung cancers. Tumor cells with high levels of HER2 have a more aggressive phenotype.

Immunohistochemical (IHC) evaluation of tumors shows that HER2 may not be expressed homogeneously among all cancer cells within the same specimen [3]. Indeed, IHC expression of HER2 as 3+ is defined as intense, complete circumferential membrane staining in more than 10% of tumoral cells [3]. Therefore, even in this best case scenario, a proportion of cells do not express HER2 on the cell membrane [3].

## Strategies to target HER2: clinical limitations

Several strategies have been developed to target HER2 including extracellular antibodies like trastuzumab which targets domain IV of the receptor and pertuzumab which binds to domain II and inhibits the heterodimerization of HER2 with other ErbB receptors; small tyrosine kinase inhibitors like lapatinib, tucatinib, or neratinib that inhibit the kinase activity; and finally, antibody-drug conjugates (ADCs) such as trastuzumab emtansine (T-DM1) which by binding to HER2 introduces a potent cytotoxic agent into HER2-overexpressing cells [4].

The first agent to reach the clinic was the anti-HER2 antibody trastuzumab given in combination with chemotherapy [4]. Subsequently, the tyrosine kinase inhibitor lapatinib was approved also in combination with chemotherapy [4]. More recently, studies have demonstrated how pertuzumab can augment efficacy when added to trastuzumab and chemotherapy [4]. Finally, T-DM1 has shown activity in patients with trastuzumab resistance [4].

In this context, disappointing results were observed with T-DM1 when compared to chemotherapy and trastuzumab in the upfront setting [5]. These results suggest that the administration of chemotherapy which targets all tumor cells irrespective of HER2 expression was key [6]. This hypothesis is supported by a recent study evaluating the results from the KRISTINE trial which showed that HER2 heterogeneity may explain the inferior outcomes of neoadjuvant T-DM1 compared to cytotoxic chemotherapy with HER2-targeted therapy [5]. An additional single arm study of neoadjuvant T-DM1 showed that response to this treatment was substantially reduced in the setting of HER2 heterogeneity [7].

## Mechanism of resistance to trastuzumab emtansine: role of novel ADCs

First generation ADCs like T-DM1 used a non-cleavable linker to bind the cytotoxic payload to the antibody in order to prevent release of the cytotoxic agent into the bloodstream and thereby reduce systemic toxicity. In this context, the activity of the payload emtansine depends on internalization and targeting of T-DM1 to intracellular sites where the ADC must suffer proteolytic degradation. Such proteolytic degradation of ADC occurs within the lysosomes where acidic proteases provoke the release of lysine-bound emtasine that may then be transported to the cytosol where it reaches its target, tubulin. If the antibody is not correctly degraded by lysosomal proteases, the activity of the compound is impaired [6]. This approach has a substantial effect on cells expressing high levels of HER2, but has limited activity on other cells with low or moderate expression [6]. To avoid this problem, second generation ADCs were developed with a cleavable linker able to release part of the payload to the extracellular environment therefore affecting non-HER2 overexpressing cells [6]. This mechanism is called bystander effect. Two examples of these compounds have reached the clinical setting with promising results.

Trastuzumab deruxtecan (DS-8201a) has an enzymatically cleavable peptide linker and a potent exatecan-derivative topoisomerase I inhibitor (DXd). This compound has activity in breast cancer cell lines with low levels of HER2 and in tumors resistant to T-DM1, probably due to the predominant effect on the population of cells with low or normal HER2 expression. The bystander effect of trastuzumab deruxtecan has permitted the development of this compound in several malignancies including tumors with low levels of HER2 [8]. Two phase I studies in breast and gastric cancer have recently shown promising results supporting further development [9]. Ongoing clinical trials include indications like urothelial carcinomas (NCT03523572) and HER2 amplified advanced gastric and gastroesophageal junction adenocarcinomas (NCT03368196). Ongoing phase 2 (NCT03248492) and phase 3 trials are evaluating trastuzumab deruxtecan in patients with HER2-positive advanced breast cancer (NCT03523585, NCT03529110) and in HER2-low breast tumors (NCT03734029). Recently, this compound received accelerated regulatory approval in the USA after showing clinical efficacy in metastatic HER2 patients pretreated with T-DM1 [10]. Of note, in a scenario where data show activity of chemotherapy-sparing regimens such as trastuzumab and pertuzumab or trastuzumab and lapatinib and with other studies ongoing in this field [11], the role of DS-8201a in some indications such as the neoadjuvant setting and particularly in the ER-positive population is an area for further study.

Another example of this kind of compounds is trastuzumab duocarmazine (SYD985). This agent includes a trastuzumab backbone and bears a cleavable linker-duocarmycin payload [12]. Experiments using patient-derived xenograft models as well as cell lines with low HER2 expression showed that SYD985 was more potent than T-DM1, in a mixed population of cell lines with a different range of HER2 expression. Similar to trastuzumab deruxtecan, this compound shows preclinical activity in low HER2-expressing cells and in heterogeneous tumors [12]. In this context, an ongoing phase III study (NCT03262935) is exploring the efficacy of trastuzumab duocarmazine in breast cancer after progression to T-DM1.

## Conclusions

The etiology of resistance to anti-HER2 therapies is heterogeneous with a key role being the upregulation of the ER signaling as an adaptative mechanism [13]. For resistance to T-DM1 specifically, HER2 heterogeneity or clonal evolution favoring lower HER2 expression appears to have an important role. The development of ADCs with cleavable linkers like trastuzumab deruxtecan or trastuzumab duocarmazine which have a bystander effect can reverse T-DM1 resistance by acting on populations of cells not overexpressing HER2. Recent studies such as Destiny 01 have shown positive results in pretreated metastatic patients [10]. ADCs with bystander effect may also be implemented more widely such as after neoadjuvant chemotherapy in patients with residual disease or in the adjuvant setting. However, in these clinical scenarios with lower tumor heterogeneity, the impact of the bystander effect may be more attenuated.

## Data Availability

NA.
